# Hyaluronan keeps mesenchymal stem cells quiescent and maintains the differentiation potential over time

**DOI:** 10.1111/acel.12567

**Published:** 2017-05-04

**Authors:** Tzyy Yue Wong, Chiung‐Hsin Chang, Chen‐Hsiang Yu, Lynn L. H. Huang

**Affiliations:** ^1^ Institute of BiotechnologyCollege of Bioscience and BiotechnologyNational Cheng Kung UniversityTainanTaiwan; ^2^ Department of Obstetrics and GynecologyNational Cheng Kung UniversityTainanTaiwan; ^3^ Department of Biotechnology and Bioindustry SciencesCollege of Bioscience and BiotechnologyNational Cheng Kung UniversityTainanTaiwan; ^4^ Institute of Clinical MedicineCollege of MedicineNational Cheng Kung UniversityTainanTaiwan; ^5^ Research Center of Excellence in Regenerative MedicineNational Cheng Kung UniversityTainanTaiwan; ^6^ Advanced Optoelectronic Technology CenterNational Cheng Kung UniversityTainanTaiwan

**Keywords:** anti‐aging, hyaluronan, lifespan, mesenchymal stem cells, quiescence

## Abstract

Hyaluronan (HA), an abundant polysaccharide found in human bodies, plays a role in the mesenchymal stem cells (MSCs) maintenance. We had previously found that HA prolonged the lifespan, and prevented the cellular aging of murine adipose‐derived stromal cells. Recently, we had also summarized the potential pathways associated with HA regulation in human MSCs. In this study, we used the human placenta‐derived MSCs (PDMSC) to investigate the effectiveness of HA in maintaining the PDMSC. We found that coating the culture surface coated with 30 μg cm^−2^ of HA (C) led to cluster growth of PDMSC, and maintained a higher number of PDMSC in quiescence compared to those grown on the normal tissue culture surface (T). PDMSC were treated for either 4 (short‐term) or 19 (long‐term) consecutive passages. PDMSC which were treated with HA for 19 consecutive passages had reduced cell enlargement, preserved MSCs biomarker expressions and osteogenic potential when compared to those grown only on T. The PDMSC transferred to T condition after long‐term HA treatment showed preserved replicative capability compared to those on only T. The telomerase activity of the HA‐treated PDMSC was also higher than that of untreated PDMSC. These data suggested a connection between HA and MSC maintenance. We suggest that HA might be regulating the distribution of cytoskeletal proteins on cell spreading in the event of quiescence to preserve MSC stemness. Maintenance of MSCs stemness delayed cellular aging, leading to the anti‐aging phenotype of PDMSC.

## Introduction

In the natural environment, stem cells reside in the stem cells niche, a microenvironment consisting of extracellular matrix (ECM) (Gattazzo *et al*., 2014), niche cells, secreted stimulants such as growth factors, chemokines, paracrines, and cytokines (Lane *et al*., 2014). The ECM is defined as a network of carbons and proteins that fill up the spaces between the cells, provide support, influence cell behaviors, cell growth, differentiation, and metabolism (Solis *et al*., 2012; Trappmann *et al*., 2012; O'Neill *et al*., 2013; Narayanan *et al*., 2014). The major component of ECM is glycosaminoglycans, large polymers that bind to ECM to produce proteoglycans (Bonnans *et al*., 2014). There are five types of characterized glycosaminoglycans: hyaluronan (HA), chondroitin, dermatan, heparin, and keratan. HA is an abundant ECM in the human body. Extrinsic signals are reported to play a role in the stem cell aging (Oh *et al*., 2014), and ECM may affect stem cell aging via extrinsic signaling, as ECM is known to influence cell quiescence in the muscle satellite niche (Thomas *et al*., 2015). ECM is vital for supporting stem cells, including keeping them functionally normal while simultaneously quiescent, throughout a person's lifetime. However, there is a lack of evidence on the role of HA in stem cell maintenance. HA is commonly known for its remarkable water retention property and is used in cosmetic products for its amazing moisturizing effects on the skin. However, it is believed that HA is much more than just a moisturizer.

Mesenchymal stem cells (MSCs) are indispensable assets for tissue engineering, and regenerative medicine. MSCs are found in various parts of the human body, including the bone marrow, brain, eyes, joints, hair follicles, reproductive organs, placenta, and umbilical cord where HA was reported to be in high content (Shiedlin *et al*., 2004). Stem cell properties including repopulation, self‐renewal (Wong *et al*., 2016), and differentiation are influenced by ECM (Gilbert *et al*., 2010; Lund & Cornelison, 2013). *In vivo*, MSCs lie quiescent unless required for repopulation or differentiation. Quiescence is a state of the reversible cell cycle arrest in which cells during which the cells reside in either G0 or G1 early phase (Martynoga *et al*., 2013).

Aging is defined as the chronological process of living units becoming old with time. At the cellular level, aging refers to the condition of the cells upon reaching their lifespan limitations with increasing hallmarks of aging such as telomere loss (Geiger *et al*., 2013), DNA damage (Sperka *et al*., 2012), reactive oxygen species accumulation (Geiger *et al*., 2013), irreversible cell cycle arrest, and tumorigenic risks. MSCs become terminally differentiated as they age (Jones & Rando, 2011). *In vivo*, MSCs can be maintained for a long period, even for a lifetime, without losing their differentiation potential, and stem cell population. Previously, aging in stem cells has been reported to be associated with ECM regulation (Wagnera *et al*., 2008; Joergensen & Rattan, 2014; Lynch & Pei, 2014). The preservation of MSCs in the natural environment is unique, and researchers have been trying to understand how nature maintains the youth of cells. Previous research on hematopoietic stem cell aging showed that stem cell aging was linked to the aging of the immune system (Geiger *et al*., 2013).

Previously, our results demonstrated that HA induced multidrug resistance through HA‐CD44 (Liu *et al*., 2009), quiescence maintenance with HA treatment (Liu *et al*., 2008), and cellular aging prevention in murine adipose‐derived stromal cells (mADSCs) (Chen *et al*., 2007). In this study, HA is confirmed to provide a positive environment to the MSCs: MSCs markers maintenance, osteogenesis differentiation potential and replicative capability preservation, reversible G0 phase maintenance, and telomerase activity maintenance.

## Results

### Hyaluronan‐coated surface maintains PDMSC morphology and MSC biomarkers expression

**Figure 1:**
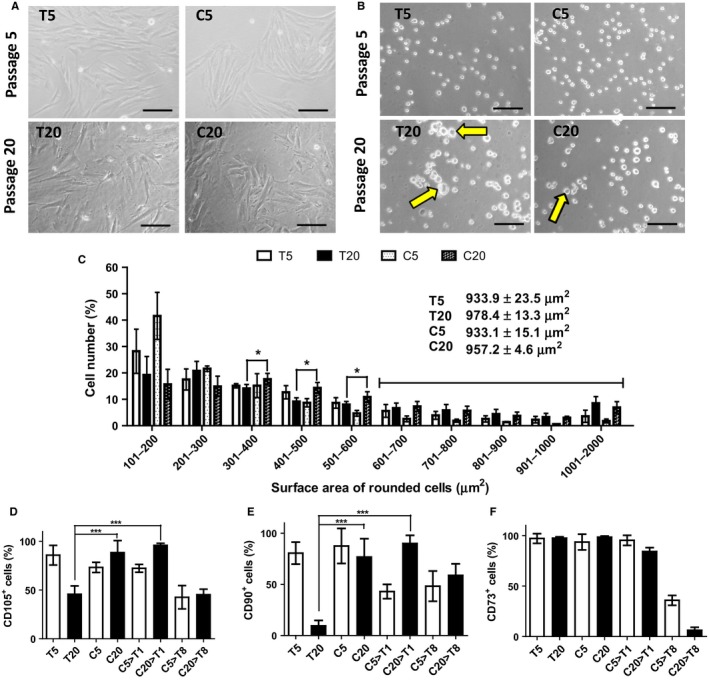
Cell morphology, size, and MSC surface marker expressions. For HA treatment, PDMSC were cultured on 30 μg cm^−2^ of HA (C) for a consecutive of four passages in young, and 19 passages in old cells. (A) Pictures showing young cell morphology at passage 5, and old cell morphology at passage 20. (B) Cell morphology when rounded‐up. Scale bar = 100 μm. (C) Cell size distribution measured from the total surface area of the rounded cells using ImageJ software. Expression of MSC biomarkers (D) CD105, (E) CD90, and (F) CD73 was determined using immunofluorescence assay. Definitions for the abbreviated culture conditions are listed in Table 1. Significant *P* values, ***P value < 0.001, **P value < 0.01, *P value < 0.05. *N* = 4.

Morphology of primary culture cells is known to become enlarged as they age during cell culture. Hence, we measured the size of PDMSC on the T and C culture conditions. The morphology of PDMSC was found to be preserved through cell size maintenance. The PDMSC were enlarged at the old passage compared to that at the young passage, and the cells on the C grew in clusters with a less spread morphology (Fig. 1A,B and Table 1). The mean size of the rounded‐up cells in the region 700–2000 μm^2^ was 978.4 ± 13.3 μm^2^, and 957.2 ± 4.6 μm^2^ for T20 and C20, respectively (Fig. 1C). The T20 had significantly lower expression of CD105 and CD90 compared to that of the C20, and C20→T1 (Fig. 1D,E). All the three MSC surface markers were consistently expressed in the C20 and C20→T1 compared to that in the T20 (Fig. 1D–F).

**Table 1:** The table explains the various groups of cells in abbreviations and their passage number

	Conditions	Passage No.	Descriptions
1	T5	5	No HA treatment cultured until P5
2	T20	20	No HA treatment cultured until P20
3	C5	5	HA treatment commenced at P2 until P5
4	C20	20	HA treatment commenced at P2 until P20
5	C5→T1	6	HA treatment commenced at P2 until P5, transferred to TCS at P6
6	C20→T1	21	HA treatment commenced at P2 until P20, transferred to TCS at P21
7	C5→T8	13	HA treatment commenced at P2 until P5, transferred to TCS at P6 until P13
8	C20→T8	28	HA treatment commenced at P2 until P20, transferred to TCS at P21 until P28

### Hyaluronan preserves osteogenesis differential potential

**Figure 2:**
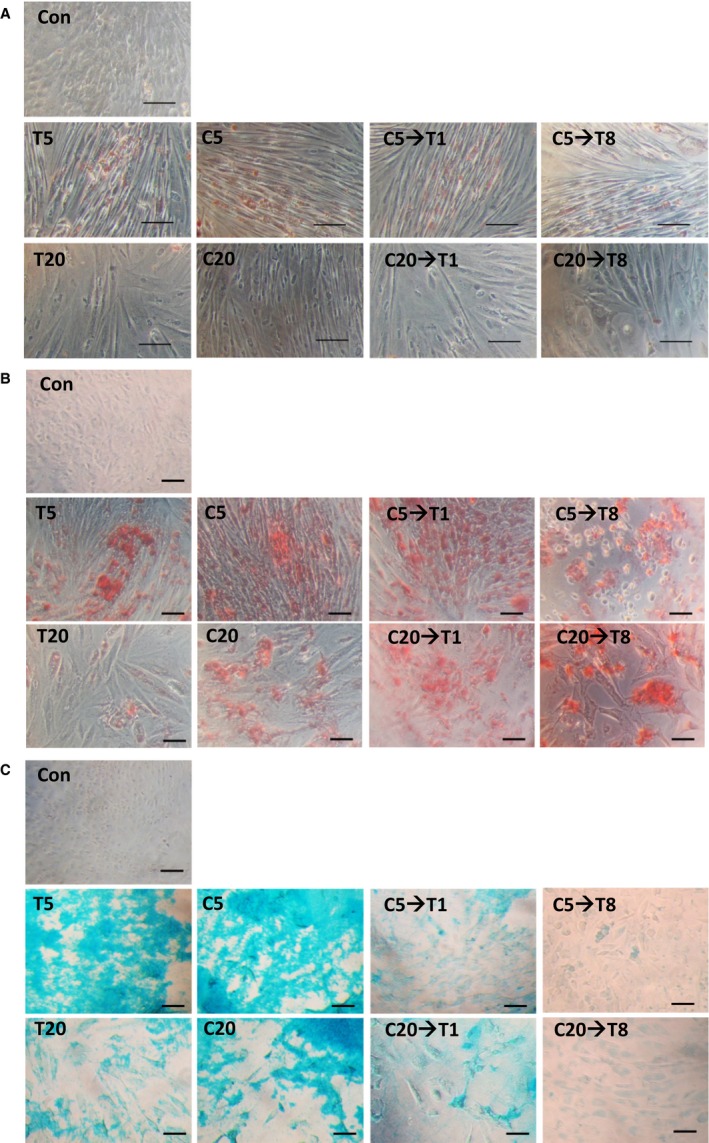
Differentiation potentials analyses for short‐ and long‐term HA treatment. PDMSC in various conditions were assessed for (A) adipogenic, (B) osteogenic, and (C) chondrogenic potentials. In adipogenesis, Oil Red O stained oil droplets red; in osteogenesis, Alizarin Red S stained calcium deposition red; and in chondrogenesis, the alcian blue stained glycosaminoglycan accumulation blue. The control groups were PDMSC, which were not given induction medium, and were observed under the microscope for the stains. Scale bar = 100 μm. *N* = 4.

MSCs possess differentiation potentials that decline with aging. The results showed that adipogenesis differentiation potential was not preserved in the old groups (Fig. 2A). Conversely, osteogenesis differentiation potential was observed in the C20, C20→T1, C20→T8, and all the young groups. However, osteogenic potential was diminished in the T20 (Fig. 2B). Regarding chondrogenesis, T5, C5, C5→T1, T20, C20, and C20→T1 had chondrogenic potential; however, it was diminished in the C5→T8, and C20→T8 (Fig. 2C).

### Cell proliferation ability maintained in the presence of hyaluronan

**Figure 3:**
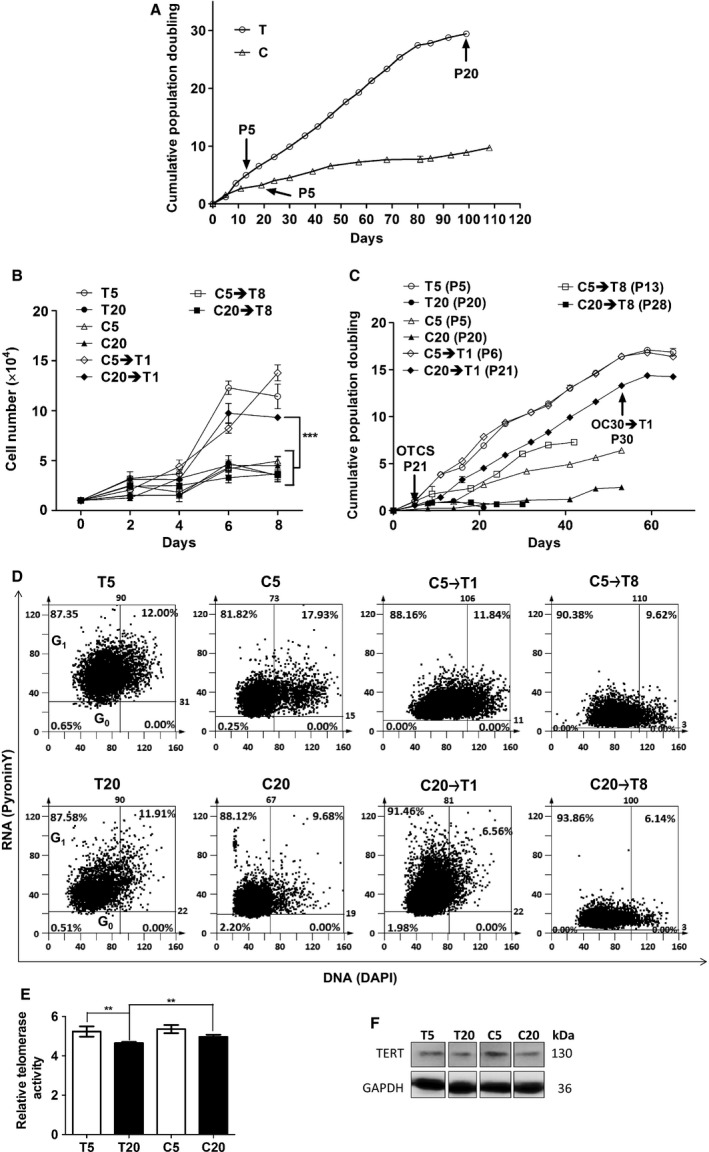
Determination of replicative capability, G0 phase cells, and telomerase activity for PDMSC with and without HA treatment. (A) Cumulative population doubling curves for T and C. (B) Growth curve derived from 8 days of culture. (C) Second cumulative population doubling curves to compare the different cell groups that commenced at various passages are shown. (D) The population of cells at G0 phase was determined using pyronin Y staining in which G0 phase cells were stained negatively for pyronin Y. (E) Telomerase is responsible for maintaining the telomere length which may influence the lifespan of the cells. Thus, the relative amount of telomeric repeats was detected using the realtime PCR. Significant *P* values, ***P value < 0.001, **P value < 0.01, *P value < 0.05. Data presented as mean ± standard deviation (Wang, Warner *et al*.). *N* = 4. (F) Telomerase reverse transcriptase, TERT protein expression level was detected using western blotting.

The replicative capability of MSCs is known to decrease during cellular aging. PDMSC grown on T proliferated only up to passage (P20) (Fig. 3A). Proliferation of PDMSC on the C was slowed down due to the quiescence of the cells (Fig. 3A). The growth curve of eight days showed that both the T5 and C20→T1 had higher proliferation rate compared to that of the T20, and were over‐confluence after 6 days. However, C5→T1 seemed to have even improved proliferation ability on Day 8 (Fig. 3B). To further understand the replicative capability, the second cumulative population curve was commenced at various passages (Fig. 3C). The results showed that the C5→T1 and T5 had identical proliferation ability, C20→T1 proliferated up to P30, whereas the T20 had completely lost proliferation ability at P21 (Fig. 3C). MSCs *in vivo* require stem cell niche that can regulate quiescence to maintain stemness. Cells at G0 phase were distinguished by pyronin Y staining. The quiescent (G0 cell cycle phase) cells with low proliferation, low mRNA manufacturing, and low protein synthesis were stained negatively (Fig. 3D). The results confirmed that both the T5 and T20 had >90% of cells that were not quiescent, whereas the C20 had 2.2%, C20→T1 had 1.98% quiescent cells during which the MSCs biomarkers, and stress fiber proteins were being detected (at 80% confluence) (Fig. 3D). Proliferation of cells is related to the telomere length that is maintained through the telomerase activity. In this study, the T20 and C20 were shown to have decreased telomerase activity compared to that of the T5 and C5; however, the T20 telomerase activity was lower compared to that of the C20 (Fig. 3E). Interestingly, the TERT protein, which controls the telomerase activity, had no significant variation between the T20 and the C20. The C5 had higher TERT protein expression compared to that of the T5 (Fig. 3F), indicating that telomerase activity was altered in the presence of HA.

### Migration ability

**Figure 4:**
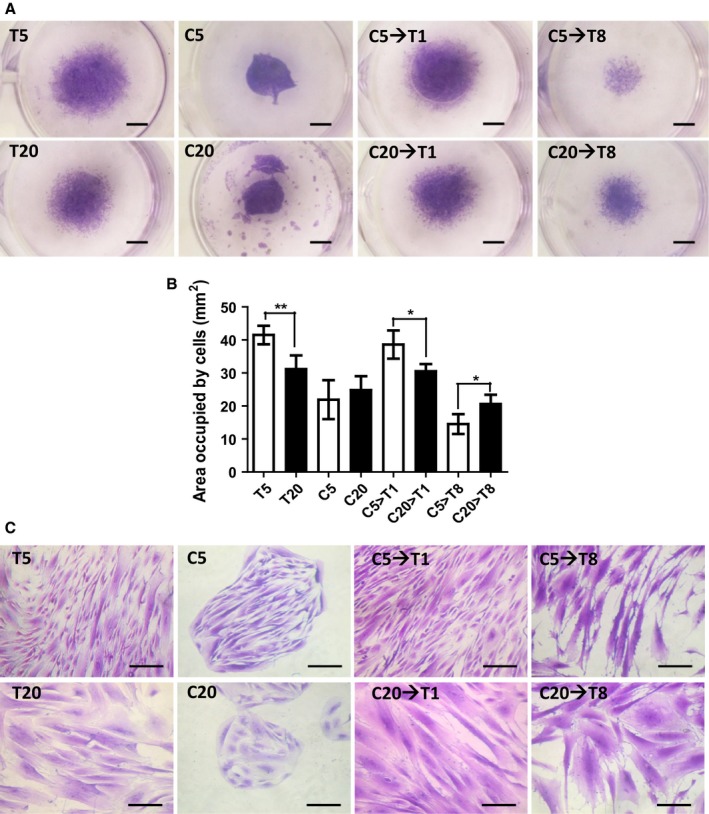
Migration ability. (A) The cells were cultured at the well center with a sterilized silicon ring placed at the center. All the silicon rings were simultaneously removed when the cells were fully attached. Migration was tested for 8 days and stained with crystal violet solution. Scale bar = 1.7 mm. (B) The migration ability was assessed by the measurement of the surface area occupied by the cells on Day 8. Data presented as mean ± SD. *N* = 3. (C) Pictures showing cells stained with crystal violet to observe the difference in morphology when migration capability was being assessed. Scale bar = 100 μm.

Migration assay showed that the T5 and C5→T1 had higher migration ability compared to that of the T20 and C20→T1 (Fig. 4A,B). Crystal violet for migration assay confirmed the enlargement of cells in the T20 when compared to that in the T5, and similar cell enlargement was observed in the C20→T1 and C20→T8 (Fig. 4C).

### Cytoskeletal proteins expression and distribution altered in the presence of hyaluronan

**Figure 5:**
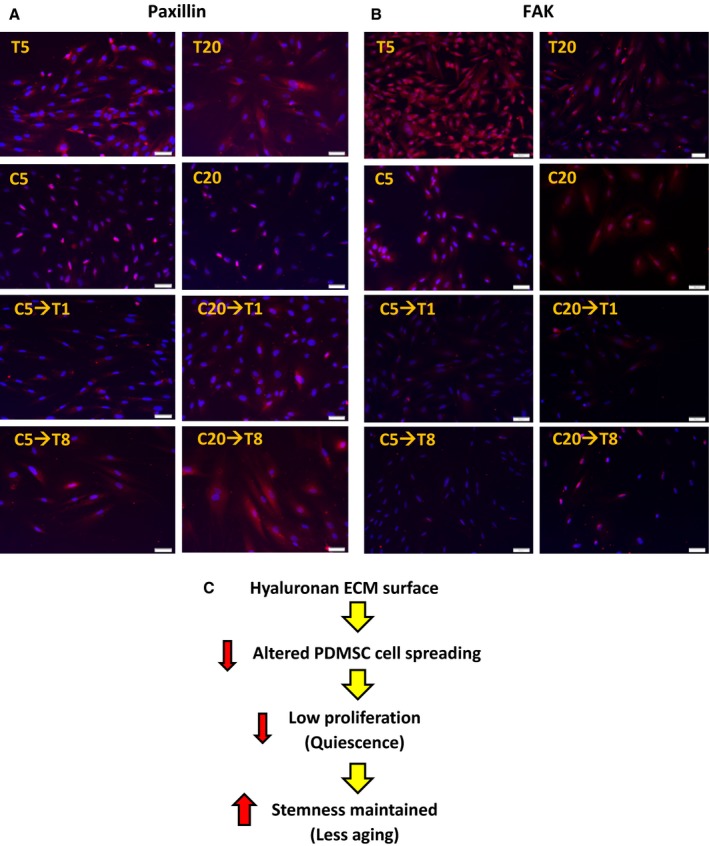
Determination of the expression of cytoskeletal proteins: paxillin and FAK. The cell morphology, spreading, adhesion, and proliferation are associated with the proteins paxillin, and focal adhesion kinase (FAK). Micrographs showing (A) FAK and (B) paxillin expressions. *N* = 3. (C) Illustration showing the effect of HA on PDMSC cell spreading that may be associated with the cytoskeletal proteins.

Cytoskeletal proteins are expressed differentially at various stages of the cells and are linked to the cell shape, adhesion, motility, and aging. The expressions of both paxillin and FAK proteins in the T5 and T20 were widely distributed throughout the cell structure, whereas in the C20→T1, the cytoskeletal proteins were distributed less in the cells which might have been caused by different cell spreading and morphology as shown earlier (Fig. 5A,B). The HA may have resulted in forming different distribution of cytoskeletal proteins; in particular, less cytoskeletal proteins were detected in the cells that had been given HA (Fig. 5C). The higher level of cell spreading as seen in the T5 and T20 might have imposed a kind of stress on the cells, thereby leading to a pro‐aging state (Fig. 5C).

## Discussion

As skin epithelial cells aged, their size became enlarged (Sokolov *et al*., 2015). Yeast cells also became enlarged as they aged that affected their cell proliferation and life‐expectancy (Yang *et al*., 2011). In the two‐dimensional cell culture, the age‐related size change was also observed in the PDMSC with the increasing passage number. The HA presence led to PDMSC growth in cluster, minimizing the change in cell size (Fig. 1A–D). MSC characterization requires several surface markers, including CD105, CD90, and CD73 (Bellayr *et al*., 2014; Ullah *et al*., 2015). The three common markers for MSCs characterization CD105, CD90, and CD73 detected in the T5, C5, and C20→T1 were higher than that in the T20 (Fig. 1E,F). *In vivo*, MSCs multipotency maintenance is possible due to stem cell niche (Okolicsanyi *et al*., 2014). Our results showed that osteogenesis differentiation potential was preserved in all the groups, except in the T20 (Fig. 2B). Although the C20, C20→T1, and C20→T8 had retained the osteogenic potential (Fig. 2B), their cumulative population doubling (PD) did not exceed that of the T20 (Fig. 3A,C). Here, it is shown that osteogenic potential is preserved through a slow‐cycling state in the C20, C20→T1, and C20→T8 (Fig. 3D). The PDMSC were quiescent on the C condition; however, they resumed proliferation once they were transferred to the T condition (C5→T1 and C20→T1), indicating that the G0 cell cycle arrest in the C was reversible. Furthermore, the C5→T8 and C20→T8 proliferation decrease was due to the transfer from the C to T condition. Our previous result showed that the osteogenic potential was preserved when the mADSC ceased to proliferate (Chen *et al*., 2007). In the study by Chen P‐Y, the mADSC on the T condition ceased proliferation after attaining four PDs, whereas the cells on the C condition attained 14 PDs with preserved osteogenic potential. Nonetheless, the loss of adipogenesis differentiation potential in the C20→T1 is possibly due to the inevitable alteration of cell morphology, and due to other undetected aging signs.

Proliferative lifespan of cells was stated to be age‐related in the human pulp cells (Shiba *et al*., 2003). The term replicative senescence is used to define the limited lifespan of the cells for *in vitro* expansion (Lloyd, 2002) which reflects the aging of the cells *in vivo*. In comparison with the T20, C20→T1 provided improved proliferation ability when considered in the same doubling time (Fig 3B,C). The PD for T was 29 on Day 99, whereas it was 9 for C on Day 99, as shown in Fig. 3A, indicating that the cells in the T condition were exhausting their proliferation ability to the identical amount of doubling time. The coated‐HA (C) required a longer time to accumulate the same number of doublings compared to no HA (T) by maintaining the cells quiescent. However, the amount of doubling time does not equal to the actual cellular lifespan; instead, the actual lifespan of the cells is when the cells have totally lost their proliferation ability. As can be seen, the total PD for PDMSC on the C transferred to T for eight consecutive passages (C20→T8) did not exceed that on only T (Figs 1 and S1, Supporting information).

Telomerase activity was detectable in all the groups (Fig. S2, Supporting information). The telomerase activity was higher in C20 compared to that in the T20 (Fig. 3D,E). A previous study showed that telomerase activity did not correlate with the telomere length maintenance in both leukemic and normal blood cells (Wang *et al*., 2005). Telomere shortening was reported to inhibit stem cell mobilization and suppressed stem cell proliferation *in vitro* (Flores *et al*., 2005). Telomerase expression was shown to prolong proliferative lifespan and maintained osteogenesis differentiation potential in the human MSCs (Simonsen *et al*., 2002). Consequently, our results showed that the TERT protein expression in the C5 was higher than that in the T5 (Fig. 3E), which explained the osteogenesis differentiation potential seen in all the old groups with HA treatment (Fig. 2B).

Crystal violet staining of the PDMSC revealed cell enlargement in the C20→T1 and C20→T8, similar to that in the T20 (Fig. 4A). The C20→T1 did not show any difference in migration ability compared to that in the T20 (Fig. 4B,C). Although the proliferative ability was preserved in the C20→T1 compared to that in the T20 (Fig. 3B,C), no difference in their migration ability was observed (Fig. 4C).

Our previous study showed that the cytoskeletal network proteins associated with cell spreading (including LIM domain, filaments, and tropomyosin) and replicative senescence‐related proteins (including Transgelin) were upregulated in the control T compared to that in the C (Wong *et al*., 2015). FAK regulates proliferation and cell cycle (Wozniak *et al*., 2004), whereas paxillin is a part of the focal adhesion proteins that regulate cell spreading through the interaction with integrin (Ramon *et al*., 2002). The FAK and paxillin were distributed throughout the cell structure of the T5 and T20 (Fig. 5A,B). Interestingly, it had been reported that stress fibers were not commonly not detected *in vivo* (Wong *et al*., 1983). This suggested that the usual two‐dimensional culture condition was not suitable for keeping stem cells. In addition, the usual two‐dimensional culture condition T showed that cells were not quiescent (T5 and T20) (Fig. 3D). We have evidence that HA reduces the formation of reactive oxygen species in the PDMSC (Solis *et al*., 2016) and increases cell viability when hydrogen peroxide (H_2_O_2_) is given to the PDMSC (Data not shown). According to a previous review by Darzynkiewicz Z and Balazs A, HA of high molecular weight inhibited the H_2_O_2_‐induced DNA damage signaling. The ATM‐H2AX pathway induced by exogenous oxidants was attenuated through the high molecular weight HA regulation. Due to this reason, HA was indeed providing an anti‐aging environment to the PDMSC (Darzynkiewicz & Balazs, 2012), and the molecular weight of HA used in this study was also high molecular weight which correlated with the previous findings.

## Experimental procedures

### Cell isolation and primary cell culture

The human placentas obtained from informed consent mothers at the National Cheng Kung University were full‐term, and the procedure for handling was approved by the Institutional Review Board. MSCs were isolated from the chorionic villi section based on a previous method. In brief, the chorionic villi tissue was excised and washed in Hanks' Balanced Salt Solution (Sigma, St. Louis, MO, USA). The tissues were minced and digested in 347 U mL^−1^ collagenase type 2 (Worthington Biochemical Corporation, Lakewood, NJ, USA) at 37 °C for 40 min. Following digestion, tissues were filtered through 500‐, 104‐, and 37‐μm filters. Mononuclear cells were further isolated by density gradient centrifugation using Percoll solution (Percoll; GE Healthcare, Uppsala, Sweden). The cells were cultured in Dulbecco's modified Eagle's medium (DMEM)–low‐glucose medium, with 10% fetal bovine serum (FBS; Gibco BRL, Life Technologies, Grand Island, NY, USA) at a cell density of 3 × 10^4^ cells per cm^2^. The cells were further subjected to adipogenesis, osteogenesis, and chondrogenesis differentiation potential characterizations after 3–4 passages.

### Cell culture with hyaluronan treatment

To prepare HA solution (HA; MW = 1560 kDa; Lifecore, Chaska, MN, USA), HA was dissolved in double distilled water and adjusted to working concentration before the experiment. For surface coating with HA, the polystyrene surface was covered with HA solution, and excess of HA solution was removed to achieve a final concentration of 30 μg cm^−2^. The plate was kept to dry for 30 min.

### Cumulative population doubling

To determine cumulative population doubling, PDMSC were seeded at 0.7 × 10^4^ cells per cm^2^ on the normal tissue culture surface without HA (T), and 2.5 × 10^4^ cells per cm^2^ on the HA‐coated surface (C) in a 24‐well plate. The cells were subcultured after reaching 80% confluence, and the number of cells was counted. The equation logarithm with base two of (final total cell number/initial cell number seeded) was used to calculate PD at every passage. Cumulative PD was the sum of all the PDs.

### Cell morphology

The cell morphology was analyzed based on the images viewed under the microscope. To measure the cell radius, the cells were fixed in 4% formaldehyde. Four repeated samples were scanned using a bright‐field microscope. The images were analyzed in the ImageJ software for the total surface area measurement.

### Immunofluorescence assay

To analyze the MSCs biomarkers, the cells were first cultured on slides coated with 1% gelatin. The next day, the cells were fixed in 4% formaldehyde for 20 min at room temperature 27 °C. Fixed cells were washed twice with phosphate buffer saline (PBS) and blocked with 1% bovine serum albumin (Sigma) for 1 h at 27 °C. The cells were incubated with primary antibodies CD105 (Abcam, San Francisco, CA, USA), CD90 (Abcam), CD73 (Abcam), paxillin (Transduction Laboratories, San Jose, CA, USA), and focal adhesion kinase (FAK; Biolegend, San Diego, CA, USA) overnight at 4 °C. Secondary antibody (IgG(H+L)TRITC purchased from Jackson Immuno Research, West Grove, PA, USA was further incubated with the cells for 1 h at 27 °C. The cells on the slides were scanned using the TissueGnostics GmbH FACS‐like Tissue Cytometry (TissueFAXS Plus) imaging system (TissueGnostics, Vienna, Austria). Images were taken for each fluorescence channel with the field of view (FOV), and the percentage of positive cells were analyzed using the TissueQuest (analysis module for immunofluorescence staining) software based on the images taken.

### Cell differentiation potential analysis

Adipogenic differentiation induction medium contained 1 μm dimethyl sulfoxide (DMSO; Sigma), 0.2 mm indomethacin (Sigma), 0.5 mm 3‐Isobutyl‐1‐methylxanthine (IBMX; Sigma), 10 μm insulin, and 10% fetal bovine serum (FBS; GibcoBRL) in DMEM–high‐glucose medium (Gibco BRL). The PDMSC were seeded at a density 1 × 10^4^ cells per cm^2^. When the cells reached 100% confluence, fresh induction medium was used to replace the old medium and changed after every 72 h. Oil Red O stain (Sigma) was used to stain the oil droplets for assessing adipogenesis. Briefly, the cells were first fixed in 4% paraformaldehyde, washed once with PBS, rinsed for 3 min with 60% iso‐propanol, and stained with Oil Red O for 1 h. After that, the cells were washed once with 60% iso‐propanol and then rinsed in double distilled water. Finally, the cells were observed under a microscope. Chondrogenic differentiation induction medium consisted of 6.25 μg mL^−1^ insulin, 50 nm ascorbic acid (JT Baker, Center Valley, PA, USA), and 10 ng mL^−1^ tumor growth factor‐beta1 (TGF‐β1; CellGS, St. Louis, MO, USA) in DMEM–high‐glucose medium without FBS. Cells were seeded at a density of 1 × 10^4^ cells per cm^2^ until 100% confluence. The medium was changed by fresh induction medium at every 72 h for 4 weeks. Accumulation of glycosaminoglycan was analyzed by staining with alcian blue (Sigma). Briefly, cells were first fixed in 4% paraformaldehyde, washed with PBS, and incubated in 1N hydrogen chloride (HCl; Sigma) solution for 5 min. Next, the cells were stained with 3% alcian blue solution in 0.1 N HCl for 30 min, washed with water, and analyzed under a microscope. Osteogenesis differentiation assay was performed using an induction medium supplemented with 10 μm DMSO, 10 nM ascorbic acid, 10 mm 2‐glycerophosphate (Sigma), and 10% FBS in DMEM–high‐glucose medium. Seeding cell density was 1 × 10^4^ cells per cm^2^, and the experiment was performed at 100% confluence. Calcification was visualized by staining the cells with Alizarin Red S (Sigma). The cells were fixed in 4% paraformaldehyde, washed with PBS, and stained in Alizarin Red S solution for 20 min. Finally, the cells were washed with water and analyzed microscopically.

### Pyronin Y staining

To analyze the G0 phase cell population, the cytochemical probe pyronin Y was used for staining the RNA. Cells were first stained with 4‐, 6‐diamidino‐2‐phenylindole (DAPI) for 30 min, washed with PBS, followed by 5 μg mL^−1^ pyronin Y at 4 °C. The next day, the cells were washed thrice with PBS and mounted on a slide using 50% glycerol. The slides were observed under the microscope. The cells on the slides were scanned using the Tissue FAXS Plus imaging system. The data were given as percentages of pyronin Y‐positive cells.

### Telomerase activity assay

To detect telomerase activity, TRAPEZE RT kit (Chemicon International, Billerica, MA, USA) was used. Procedures were performed according to the manufacturer's instructions. First, protein lysates were collected and lysed in 3‐[(3‐cholamidopropyl) dimethylammonio]‐1‐propanesulfonate (CHAPS) lysis buffer. The lysed proteins were quantified using the Bradford assay and stored at −80 °C. To quantify the telomeric repeats, 5 μL of 5× RT reaction buffer with Amplifluor primers and 0.4 μL (2 U) of *Taq* polymerase were added to the protein lysate containing 5000 cells. The final volume of each reaction was adjusted to 25 μL by PCR grade water. Before performing realtime PCR, the samples were preheated at 30 °C for 30 min. After that, realtime PCR (LightCycler^®^ 480 Real‐Time PCR instrument; Roche, Rotkreuz, Switzerland) was performed with the following conditions: 95 °C for 2 min, 1 cycle; 94 °C for 15 s, 59 °C for 1 min and 45 °C for 10 s, 45 cycles. For the standard curve, known concentrations of the standard telomeric repeats, TSR8, were used: 20 amoles μL^−1^, 2 amoles μL^−1^, 0.2 amoles μL^−1^, and 0.02 amoles μL^−1^. The linear equation derived from the standard TSR8 concentrations was used to calculate the relative amplified telomeric repeats in the experimental samples. The amplified PCR products were separated on a 12% nondenaturing polyacrylamide gel at 60 V for 1.5 h. For visualization, the gel was stained with ethidium bromide (Sigma) for 20 min, exposed to UV light (UV Light Box, Taipei, Taiwan Patent No. 153495), and image taken by DigiGel Image System (Topbio, Taipei, Taiwan). The representative gel image is provided in the Fig. S2 (Supporting information).

### Western blotting

Protein lysates collected from the cells using radioimmunoprecipitation assay (RIPA) buffer were denatured and resolved on an 8% polyacrylamide gel and transferred onto a polyvinylidene fluoride (PVDF) membrane. The blots were blocked in Tris‐buffered saline with Tween 20 (TBST) with 5% skim milk for 1 h, followed by incubation with the primary antibodies, telomerase reverse transcriptase, TERT (TA300468), and GAPDH (GTX100118) overnight at 4 °C. Then, the primary antibodies were removed, washed with TBST, and then incubated with the secondary antibodies, goat anti‐rabbit IgG(H+L)‐peroxidase. The protein bands were visualized by ECL bioluminescence method, and the images were taken using the IVIS50 imaging system (Caliper Life Sciences, Hopkinton, MA, USA).

### Cell migration assay

To detect the variation in cell migration, 1 × 10^4^ cells were cultured in a silicon ring placed at the center of a 24‐well plate. Once the cells were attached, the ring was removed, and the cells were left to migrate for 8 days. imagej software, Bethesda, MD, USA was used to measure the total surface area covered by the migrated cells from the center.

### Statistical analysis

For the statistical analysis, Student's *t*‐test was used to calculate the *P* value.

## Funding

This research was supported by the MOST 105‐2622‐8‐006‐010‐TB1 and MOST 105‐2119‐M‐006‐017 grants from the Ministry of Science and Technology in Taiwan.

## Author contributions

The experimental conception and design were performed by Professor Lynn L. H. Huang. All experiments were performed in her laboratory, including financial and administrative support, resources and intellectual support, supervision of the experiments that contributed to edition, revision, and final approval of the manuscript. The experiments were performed, and the manuscript was written by her PhD student Tzyy Yue Wong. The placentas of patients were provided by Professors Chiung‐Hsin Chang, and Chen‐Hsiang Yu.

## Conflict of interest

The authors of this research declare no financial conflict of interest at the moment. However, the related works of stem cells on hyaluronan‐coated surface were patented as: 1) US Patent 12/155.487; 2) Taiwan Patent I473879; 3) US Patent 8426196; and 4) Taiwan Patent I465567.

## Supporting information


**Fig. S1**. Total population doubling for PDMSC after long‐term HA treatment.
**Fig. S2**. Representative gel image for TRAP assay showing telomerase activity in the PDMSC.
