# Supplementary material for: Hyaluronan keeps mesenchymal stem cells quiescent and maintains the differentiation potential over time
Source: Aging Cell. 2017 May 4;16(3):451–60. doi: 10.1111/acel.12567 (PMC5418204; doi:10.1111/acel.12567)
Supplement: Supplementary file 1 — Fig. S1. Total population doubling for PDMSC after long‐term HA treatment. Fig. S2. Representative gel image for TRAP assay showing telomerase activity in the PDMSC. [file ACEL-16-451-s001.pptx]

## Slide 1
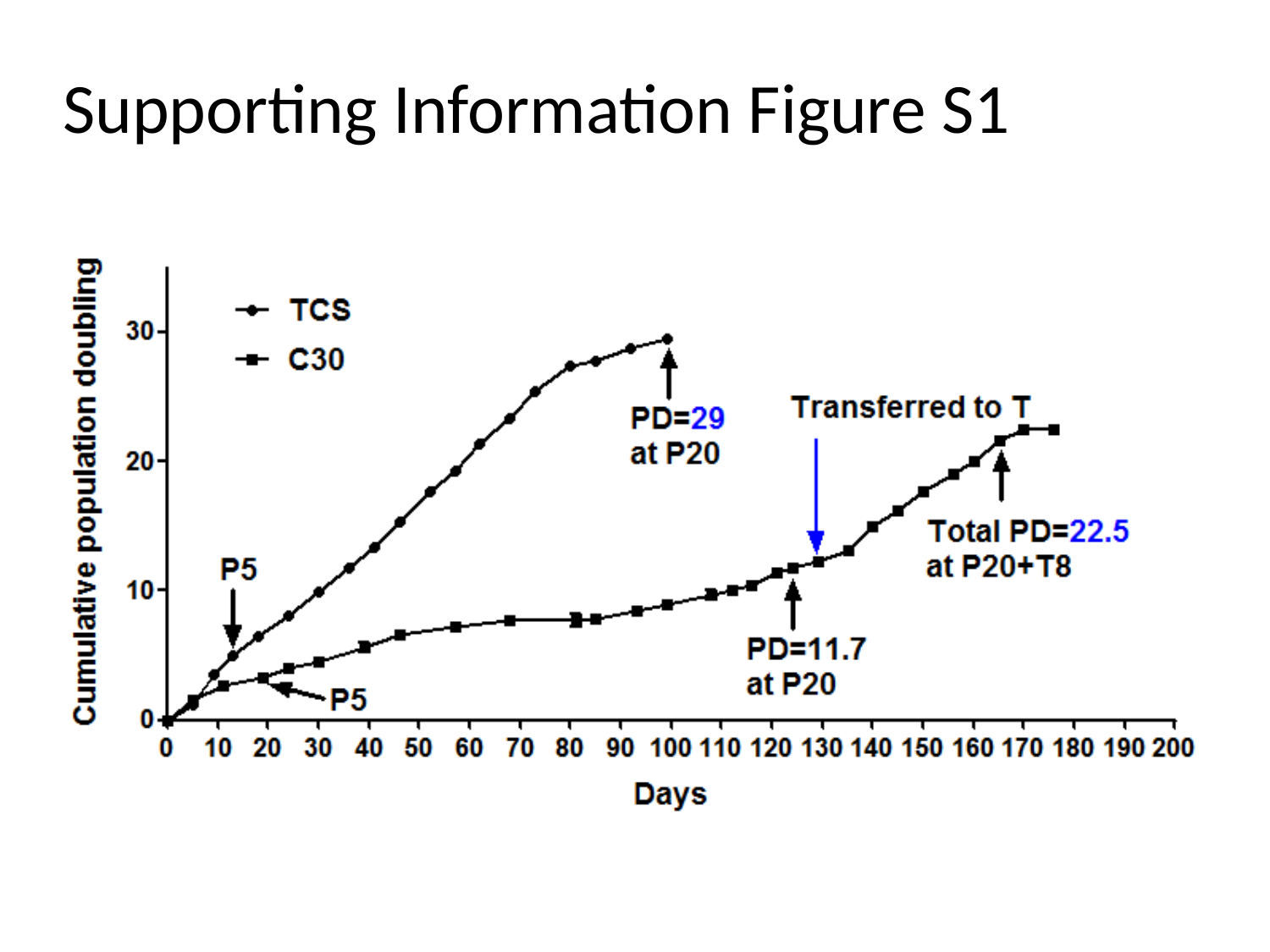

Supporting Information Figure S1

## Slide 2
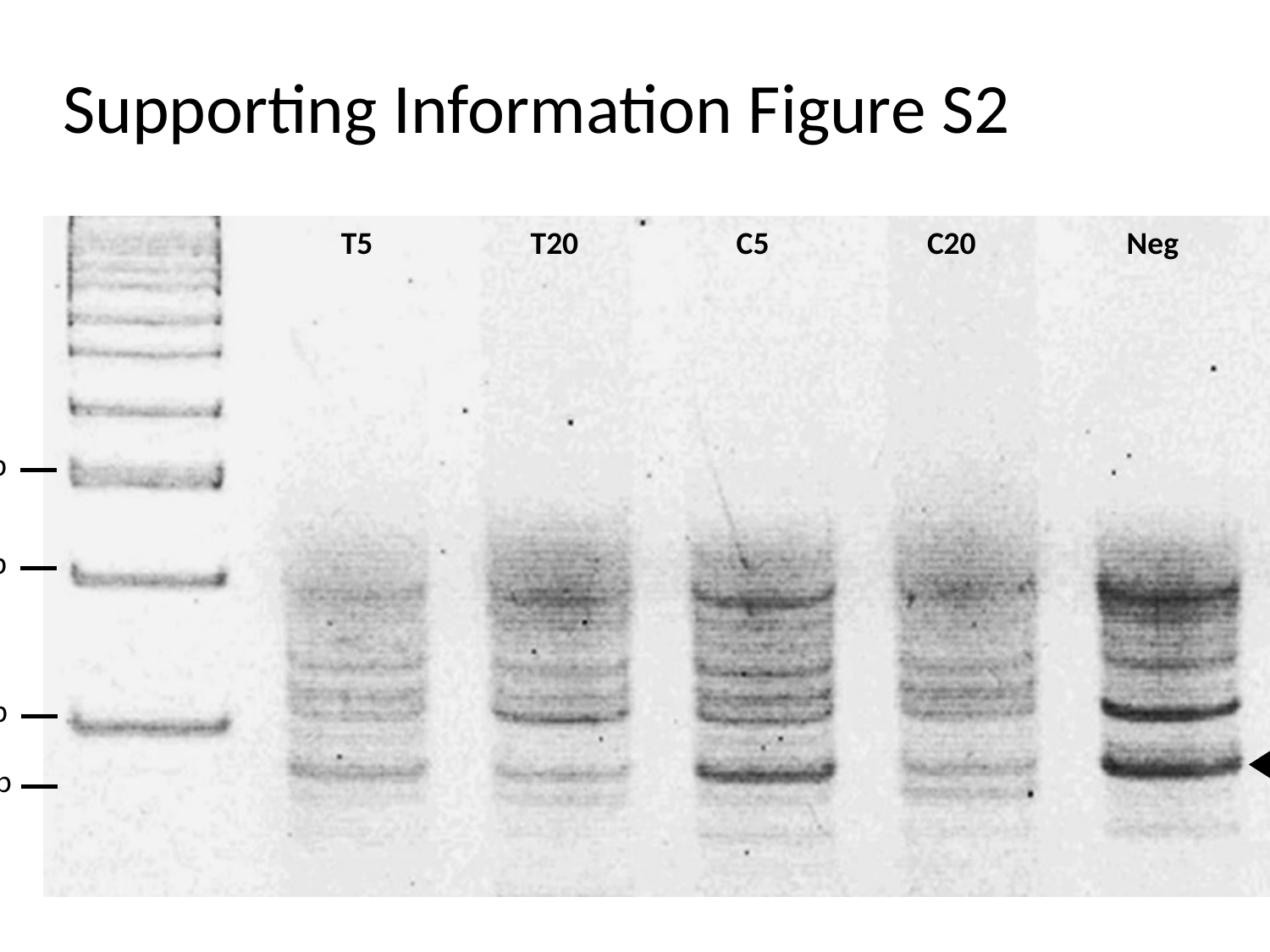

Supporting Information Figure S2
T5 T20 C5 C20 Neg
200 bp
150 bp
100 bp
IC
83 bp

## Slide 3
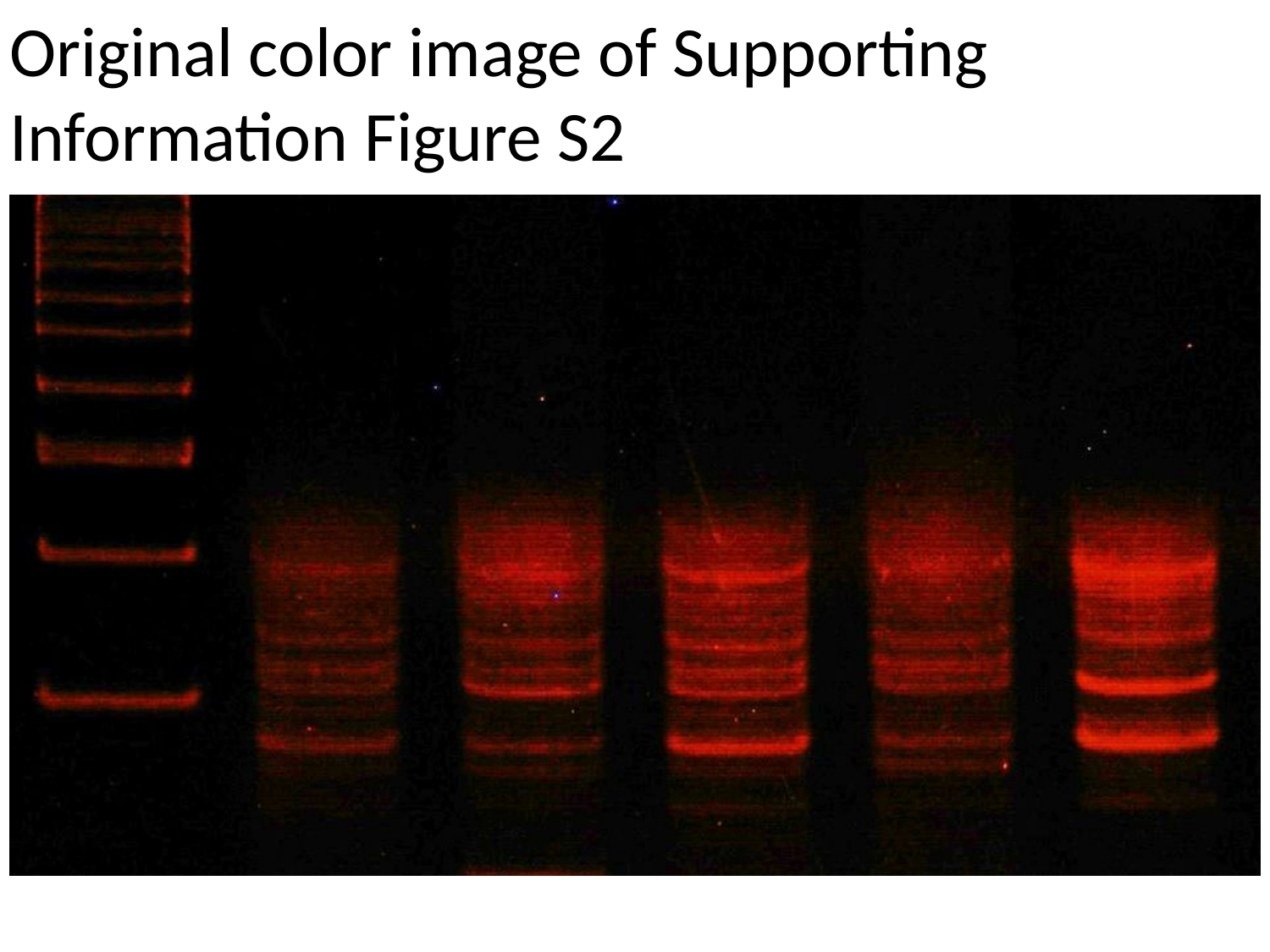

Original color image of Supporting Information Figure S2
